# Impedance Enhancement of Textile Grounded Loop Antenna Using High-Impedance Surface (HIS) for Healthcare Applications

**DOI:** 10.3390/s20143809

**Published:** 2020-07-08

**Authors:** Mohammed M. Bait-Suwailam, Isidoro I. Labiano, Akram Alomainy

**Affiliations:** 1 Department of ECE, Sultan Qaboos University, Muscat 123, Oman; 2 Department of EECS, Queen Mary University of London, London E1 4NS, UK; i.ibanezlabiano@qmul.ac.uk (I.I.L.); a.alomainy@qmul.ac.uk (A.A.)

**Keywords:** high-impedance surface, loop antenna, textile sensor, wearable sensor

## Abstract

In this paper, impedance matching enhancement of a grounded wearable low-profile loop antenna is investigated using a high-impedance surface (HIS) structure. The wearable loop antenna along with the HIS structure is maintained low-profile, making it a suitable candidate for healthcare applications. The paper starts with investigating, both numerically and experimentally, the effects of several textile parameters on the performance of the wearable loop antenna. The application of impedance enhancement of wearable grounded loop antenna with HIS structure is then demonstrated. Numerical full-wave simulations are presented and validated with measured results. Unlike the grounded wearable loop antenna alone with its degraded performance, the wearable loop antenna with HIS structure showed better matching performance improvement at the 2.45 GHz-band. The computed overall far-field properties of the wearable loop antenna with HIS structure shows good performance, with a maximum gain of 6.19 dBi. The effects of bending the wearable loop antenna structure with and without HIS structure as well as when in close proximity to a modeled human arm are also investigated, where good performance was achieved for the case of the wearable antenna with the HIS structure.

## 1. Introduction

Wearable sensors have received much attention in recent years and are becoming common and most desirable in lots of consumer-related electronics, due to their ease of integration with fabrics and electronic components and circuits, flexibility and conformability [1,2,3]. With particular emphasis on biological related applications, such devices, including wearable sensors (antennas), are capable of sensing, monitoring and wirelessly interconnecting with other nearby wearables in what is known as body-centric networks [4,5,6]. However, performance of such wearable radiators in terms of impedance matching, gain, efficiency, and roboustness may severely degrade when in close proximity to human body or tissues depending on number of factors including, but not limited to, deployed antenna and design complexity, nature of textile-based materials, channel losses within human body tissues. This in turn poses challenges in the design, development and reliability of wearable antennas for body-centric healthcare communications [7,8].

Despite such challenges, wearable radiators with metallic reflectors are favourable in many healthcare applications, for instance, for sensing, monitoring, and detection of abnormalities in human tissues and at the same time reducing significant electromagnetic radiation exposure to human tissues and consequently reducing effects of specific absorption rate (SAR). In other words, reducing impedance mismatch of wearable antennas with ground plane when placed in close proximity to human body [9]. However, such metallic reflectors result in performance degradation of wearable low-profile antennas, depending on the deployed antenna design, textile fabric and how far the wearable radiator is from the metallic reflector. From image theory point of view, this performance degradation comes in place due to the destructive interference between the main radiator and its image, being 180${}^{\circ }$ out of phase. In practice, this degradation can be alleviated in one way via increasing textile’s thickness, however, this puts a constraint in size and height of wearable textile solutions for healthcare applications.

Amongst the widely known techniques that are advantageous to antenna systems and subsystems, including low-profile antennas, is the use of electromagnetic bandgap structures, also known as high-impedance surfaces, (HIS) [10,11]. Such structures had been adopted in the past in many rigid-based, non-fabric, antenna structures in order to improve antennas operational matching impedance and/or enhance the far-field properties [12,13]. Further, in recent years, high-impedance surfaces for enhancement of wearable antennas were adopted in literature [14,15,16,17,18]. In previous HIS related attempts for wearable radiators, tremendous efforts were mainly focused on microstrip patch antennas or dual-band operational patch antennas, for instance, the works in [16,18]. However, to the best of our knowledge, the impact of such engineered structures on wearable grounded loop antennas has not yet been extensively investigated. Furthermore, performance assessment of wearable loop antennas as a function of variant textile properties and types as well as antenna geometry need a revisit. This is in addition to quantifying performance of such wearable loop antennas due to bending effects, impact of metallic reflectors and in close proximity to human tissues.

The contributions of this paper are: to comprehensively investigate, numerically and experimentally, effects of several textile properties and parameters on the performance of wearable loop antenna. Furthermore, one more aspect that this paper aims to explore is the development of low-cost conductive tape based-textile sensor as an effective manufacturing technique for metallic layers of a multi-layer based textile antenna instead of the use of conductive textile yarns in computer-aided embroidery [1,19]. We believe that the use of conductive adhesive tapes will overcome some of the challenges that embroidery manufacturing technique may encounter for development of multilayer antenna structure. As far as the potential applications of the developed textile sensor with embedded HIS structure are concerned, wearable antenna structure can be adopted in applications centered around wearable healthcare for sensing and detection purposes. The antenna needs to be low-profile with low emitted radiated power and still needs to maintain reasonable performance. In this work, we demonstrate impedance enhancement of grounded textile loop antennas using carefully constructed high-impedance structure, in which the overall wearable structure is maintained light weight, conformal, and low in profile. Numerical modeling and full-wave simulations were carried out using CST Microwave Studio (CST MWS) and experimental validations are also presented. In order to demonstrate the integrity and robustness of this wearable loop antenna with HIS structure, numerical studies to address the effect of the antenna performance when placed close to the human arm are presented.

The rest of the paper is organized as follows. Section 1 presents the wearable loop antenna design and investigates the effects of several textile parameters on the performance of a wearable loop antenna, taking into consideration as well as loop antenna geometrical parameters. Section 2 then presents the application of impedance enhancement of a grounded wearable loop antenna using HIS structure in order to tackle the problem of wearable antenna’s close-proximity to a metallic reflector. Far-field properties are also presented for the wearable loop antenna with and without the HIS structure. The effects of bending and on-body contact are also investigated. Section 3 concludes with findings of this research work.

## 2. Effects of Textile Properties on Wearable Loop Antenna Performance

**Figure 1:**
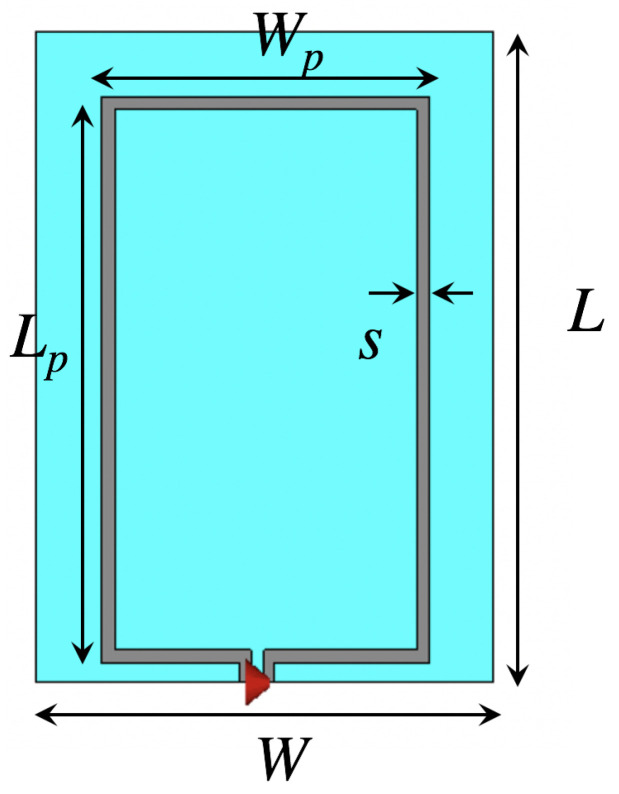
Top view of the modeled textile loop antenna along with its dimensions.

Figure 1 depicts the modeled wearable loop antenna structure that is printed in a very thin non-grounded 0.242 mm -thick textile cotton fabric, where the loop antenna is of size L × W = 50 mm × 35 mm, respectively, designed to resonate around 2.45 GHz, being a suitable candidate for healthcare applications. The metallic loop of size $L_{p}$ × $W_{p}$ = 43.5 mm × 25.1 mm, respectively, metal trace width s = 1 mm was used. In the numerical full-wave simulations, the metallic loop was edge-fed using an ideal lumped port. The performance of the wearable loop antenna was measured through vector network analyzer, where a 50-Ω SMA connector was soldered to the loop’s edges. Several materials had been adopted in literature to construct the conductive parts of textile wearable antennas, including computerized based embroidery manufacturing techniques using conductive threads [20] and conductive inks [21,22,23]. In this paper, a low-cost adhesive conductive tape was considered for the manufacturing of the multi-layer-based antenna structure, i.e. construction of the metallic loops as well as the metallic patches of the HIS structure. In order to maintain accuracy in geometry of metallic layers of the wearable sensor as well as the HIS structure, a precise blade cutter machine (Graphtec Craft-Robo CC300, Graphtec Corporation, Yokohama, Japan) from Materials Engineering Laboratory at Queen Mary University of London was used. The metallic layers were then manually attached with a thin layer of acrylic pressure-sensitive conductive adhesive with good heat resistance to the textile substrates.

**Figure 2:**
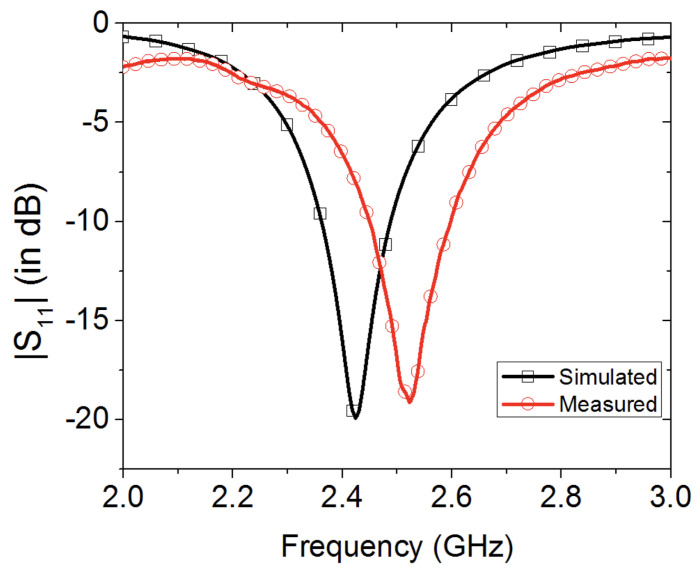
Simulated versus measured reflection coefficient of the wearable loop antenna with loop metallic width of s = 1 mm on textile cotton fabric with permittivity of 1.2, loss tangent of 0.02 and thickness of 0.242 mm.

**Figure 3:**
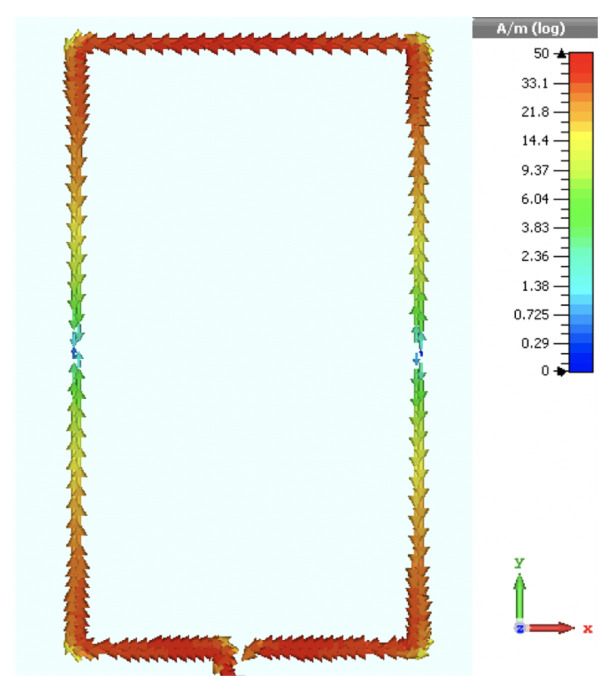
Snapshot of computed surface current distribution for the wearable loop antenna.

Figure 2 shows the numerically computed and measured reflection coefficient of the wearable loop antenna that is presented in Figure 1. A minor discrepancy was noted between the simulated and measured data, where a slight shift was observed in the antenna’s resonance frequency from 2.42 GHz (simulated case) to 2.52 GHz (fabricated prototype). This is attributed mainly to the adoption of only estimated figures for the thickness and electrical properties of the textile cotton fabric. Note that an adhesive copper tape was used for the metallic loop. A snapshot of the electric surface current distribution for the wearable antenna at a frequency of 2.42 GHz is displayed in Figure 3.

Next, effects of several textile parameters on the performance of the wearable loop antenna are investigated in this paper, namely: (A) textile fabric type and loop metallic width, (B) textile fabric electrical properties, considering the electrical properties and thickness variation, (C) the effects of backing wearable loop antenna with a metallic reflector, (D) bending effects, and (E) the performance of the wearable loop antenna with and without the HIS structure when in close proximity to a human arm.

### 2.1. Effects of Textile Fabrics Type and Loop Geometry

**Figure 4:**
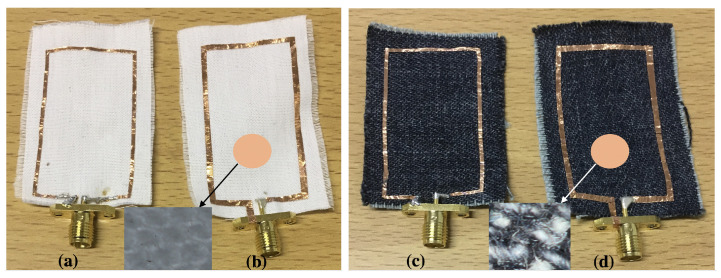
Fabricated wearable loop antenna on various textile fabrics: (**a**) in cotton with width = 1 mm; (**b**) in cotton with width of 2 mm; (**c**) in jeans with width = 1 mm; and (**d**) in jeans with width of 2 mm. Insets provide a zoomed-in microscopic view of fabrics.

In this section, the effects of textile fabrics are studied experimentally, where two types of textile fabrics are considered: cotton and jeans (wash cotton), with different metallic widths of 1 mm and 2 mm. Images of the fabricated wearable loop antenna that is printed on various textile fabrics are presented in Figure 4.

The characterization of materials electrical properties has extensively been conducted in literature, including textile fabrics [24,25,26,27,28]. The electrical properties of the studied textile fabrics in this paper are as follows: cotton fabric with electrical relative permittivity of 1.5 and thickness of 0.242 mm, and jeans (wash cotton) fabric with relative permittivity of 1.67 and thickness of 0.75 mm. Such electrical properties are adopted since they had already been measured and validated in [27], while thicknesses of the two fabrics were estimated in the laboratory using a thickness Gauge.

**Figure 5:**
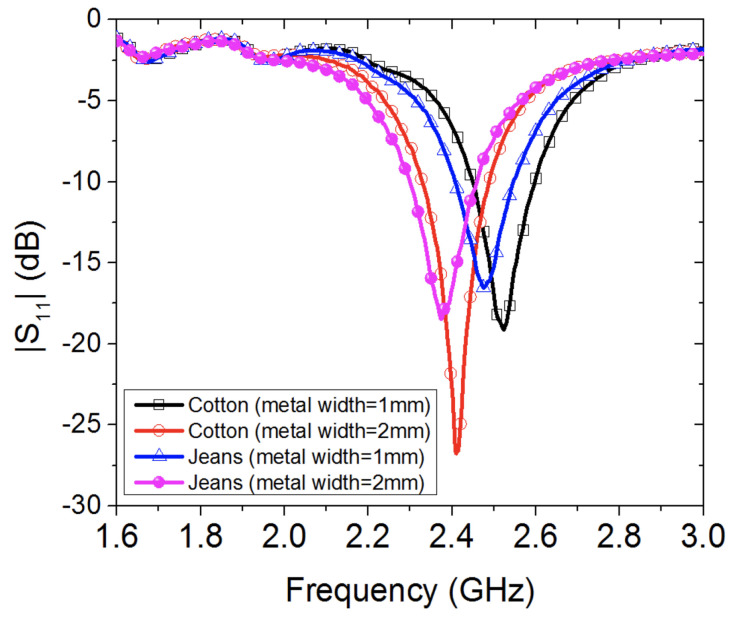
Measured reflection coefficient of the wearable loop antenna with two different fabrics: cotton and jeans, and two different metallic widths of 1 mm and 2 mm.

Figure 5 shows the measured reflection coefficient of four textile wearable loop antenna prototypes (each textile fabric with two different metallic widths, see Figure 4). For the textile cotton fabric loop antennas, it was observed that increasing the metallic width of the wearable loop antenna results in shifting the resonance frequency to lower frequencies, for instance here, from 2.517 GHz to 2.413 GHz resulting in a 4% shift in resonance frequency. Similar trend was also observed for the textile jeans fabric antennas, where the resonance frequency was shifted from 2.474 GHz to 2.378 GHz resulting in a 3.8% shift in resonance frequency. For the wearable loop antennas with metallic width of 2 mm, a strong resonance dip was noticed for the textile cotton fabric loop antenna as compared against textile jeans fabric antenna.

### 2.2. Effects of Textile Fabrics Properties

The effects of both electrical properties and thickness of textile fabrics on the performance of wearable loop antenna are investigated numerically. The effect of the textile substrate’s electric permittivity is considered first through numerical parametric study by varying the real part of the textile fabric relative permittivity from 1.2 to 2.0, while fixing the textile substrate’s loss tangent at 0.02 and the textile fabric’s thickness as 0.242 mm for cotton fabric and 0.75 mm for jeans fabric.

**Figure 6:**
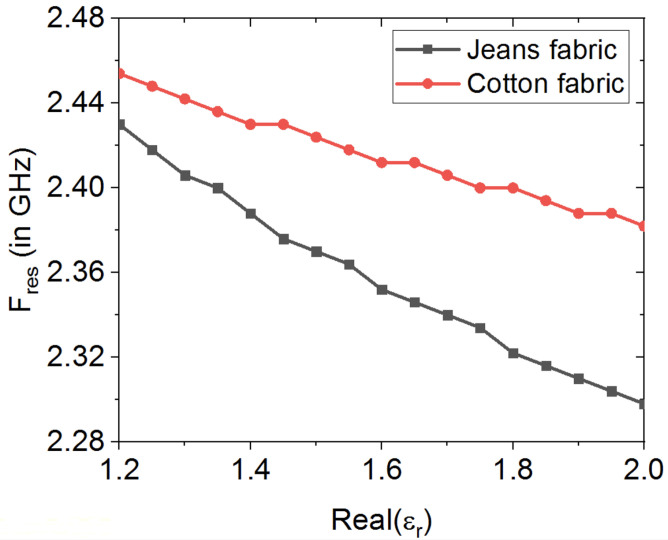
Numerical study on effect of textile fabrics’ electric permittivity on loop antenna’s resonance frequency.

Figure 6 shows the numerically estimated loop antenna’s resonance behavior, as the electric permittivity is increased. As expected, the antenna’s resonance tends to shift to lower frequencies, as the permittivity is increased, which is attributed to the increased capacitance of the host textile substrate and hence results in lowering the wearable sensor’s resonance frequency. It is also observed that for real-part of permittivities >1.4, the antenna’s resonance tends to shift to lower frequencies in a much faster rate for jeans fabric as compared against the antenna’s resonance behavior for the cotton fabric. For instance, at a permittivity of 1.8, the resonance frequency of the textile jeans-based loop antenna is at around 2.32 GHz, while that for textile cotton-based loop antenna is at 2.39 GHz.

**Figure 7:**
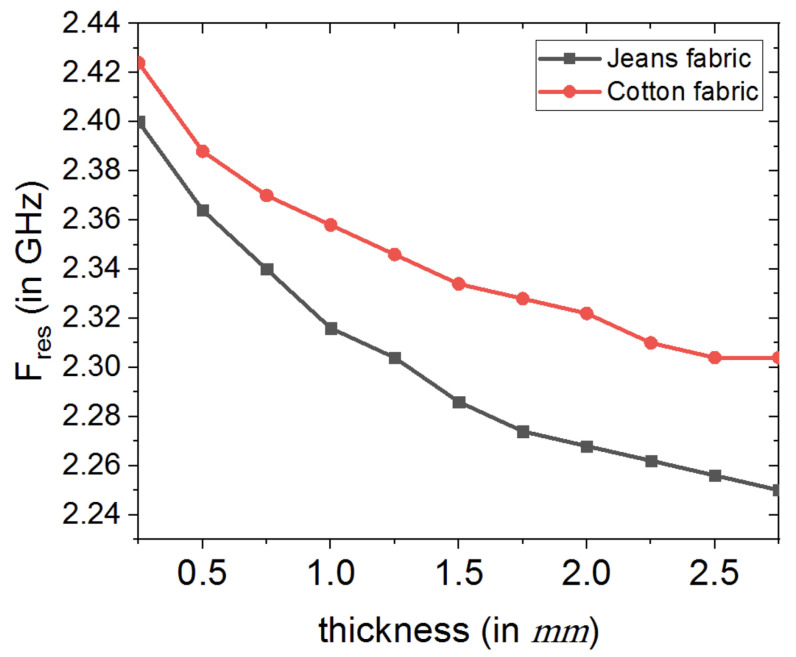
Numerical study of the effect of textile fabrics’ thickness on loop antenna’s resonance frequency.

Figure 7 depicts the dependence of antenna’s resonance frequency on the thickness of textile fabrics substrate, while fixing the electric permittivity of the two textile fabrics. In this study, variation of the textile fabric thickness is considered from 0.25 mm to 2.75 mm. From this numerical parametric study, it is shown that as the thickness of the textile fabric substrate is increased over 0.75 mm, faster drop in loop antenna’s resonance frequency is incurred for jeans fabric-based antenna as compared against the cotton based loop antenna.

**Figure 8:**
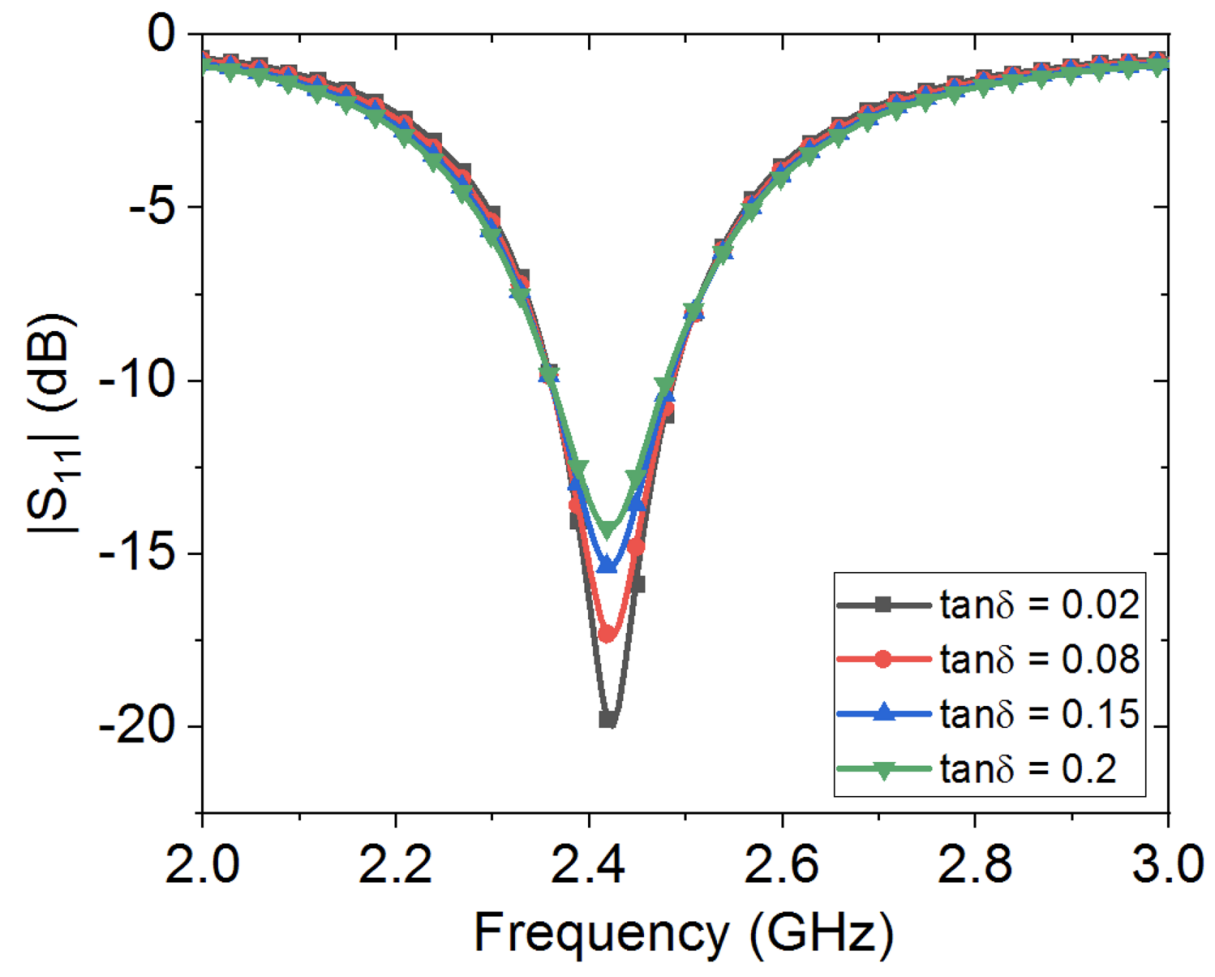
Numerical study of the effect of textile cotton fabric electrical loss, tanδ, on wearable loop antenna’s resonance frequency.

Figure 8 shows the effects of textile electrical loss, tanδ, on the loop antenna’s performance. In this study, cotton fabric is considered, where tanδ is varied from 0.02 to 0.2. It is observed that the loop antenna’s dip strength is decreased as the loss is increased. For instance, at a loss tangent of 0.08, the antenna’s dip strength is at −17.33 dB, while at a loss tangent of 0.15, the antenna’s dip strength is around −14.23 dB.

### 2.3. Bending Effects

It is essential that the wearable antenna maintains its robustness while in bending conditions or with body tissue contacts. Due to the high lossy dielectric contrasts of body tissue layers, it may occur that resonance frequency of the wearable antenna gets shifted from its principal frequency of operation, depending on the employed textile fabric and its thickness. In this section, experimental studies are carried out in order to investigate the effects of bending on the wearable loop antenna, where two bending fixtures were used: (i) a semi-cylindrical foam (with $\epsilon_{r}$ nearly 1.05) with a radius of 20 mm, (ii) and a cylindrical fixture made of PVC with a radius of 37.5 mm.

**Figure 9:**
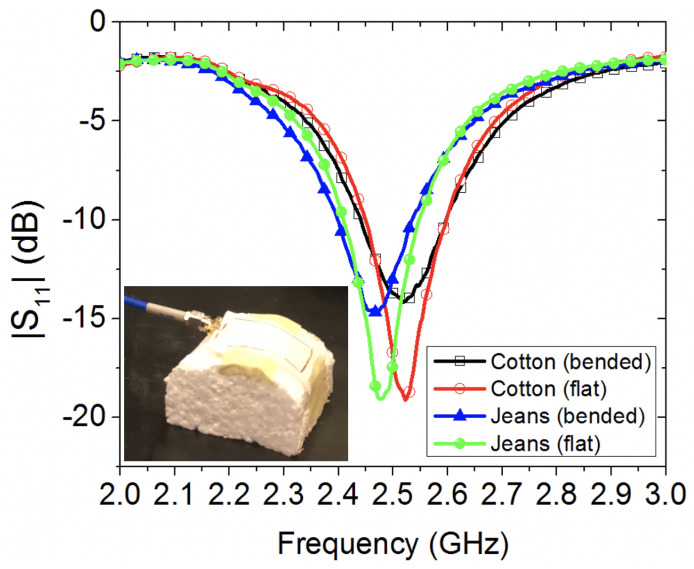
Experimental study of the effect of bending two wearable loop antenna prototypes, on a semi-cylindrical foam fixture.

Figure 9 depicts the effects of bending on two textile wearable loops when bent on a semi-cylindrical foam fixture. Comparison is made against the non-bending scenarios (i.e., flat wearable loop antennas). As expected, antennas’ resonance dip did not change for both prototypes, since the bending body is almost as close to free-space, except for a slight reduction in resonance level, which is resulted from the disturbance of current distribution while on bending mode.

**Figure 10:**
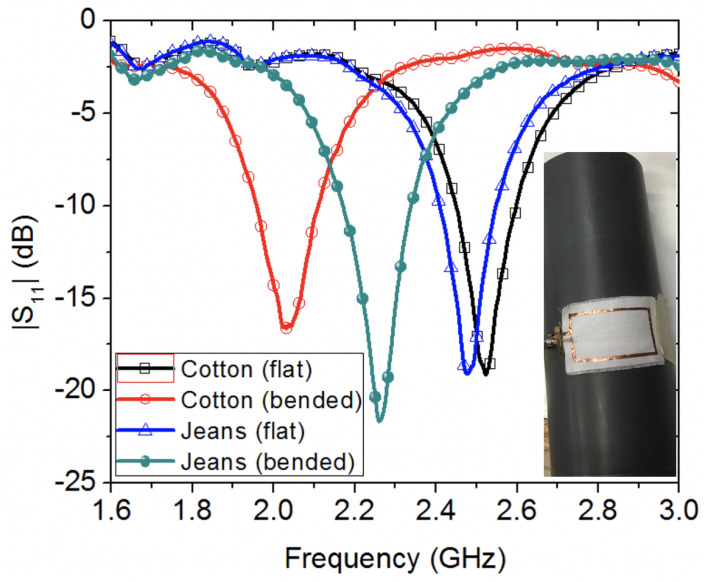
Experimental study of the effect of bending two wearable loop antenna prototypes, oriented perpendicular to the longitudinal axis of a cylindrical PVC fixture.

**Figure 11:**
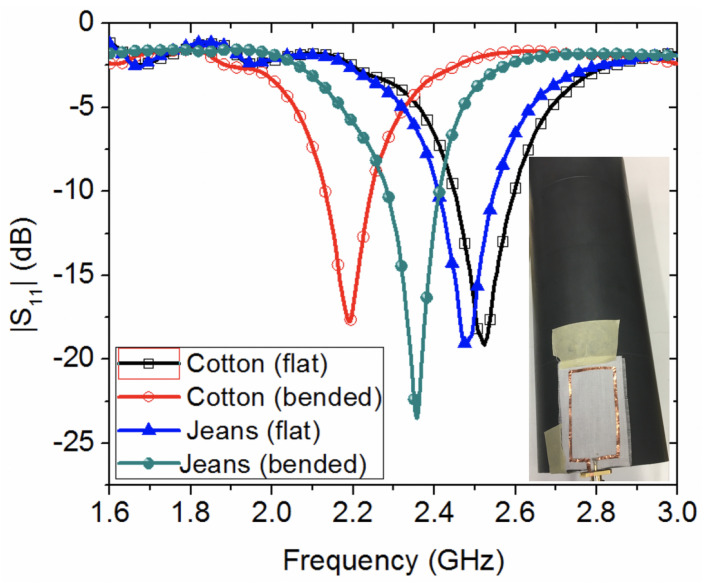
Experimental study of the effect of bending two wearable loop antenna prototypes, oriented parallel to the longitudinal axis of a cylindrical PVC fixture.

Figure 10 shows the reflection coefficient results when bending the loop antennas, oriented perpendicular to the longitudinal axis of a cylindrical fixture, made of PVC with a radius of 37.5 mm, while Figure 11 shows the case of bending oriented parallel to the longitudinal axis of the PVC fixture, as shown in the insets of both Figure 10 and Figure 11. From the studied bending scenarios, it is observed that bending the wearable loop sensor has resulted in significant shift to the resonance frequency for the scenario in Figure 10 as compared against the bending scenario in Figure 11. This is attributed to two main effects. Firstly, the orientation of bending of the wearable sensor is different. Secondly, the encountered loading effect of PVC electric permittivity of and its associated loss.

### 2.4. Effects of Backing Wearable Loop Antenna with Metallic Reflector

According to the principle of image theory, performance of low-profile radiators when placed in close proximity to metallic reflectors is compromised and may severely degrade when radiator is placed very close to the metallic reflector, due to the destructive interference between the original radiator and its image (i.e., 180${}^{\circ }$ phase difference). In other words, when radiator is placed on top of a metallic reflector with a thickness of much less than λ/4, where λ is the operating wavelength, performance degradation is incurred. In this section, a comparison between textile loop antenna with and without a metallic reflector is analysed both numerically and experimentally.

**Figure 12:**
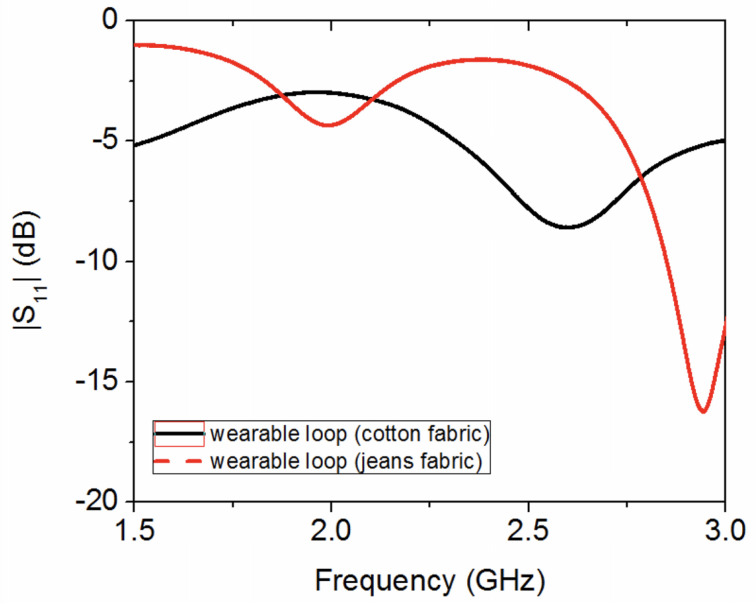
Measured reflection coefficient for two textile wearable loop antennas with metallic ground sheets.

As can be seen from Figure 12, the resonance of both wearable loop antenna prototypes disappears, due to the presence of the metallic ground plane.

**Figure 13:**
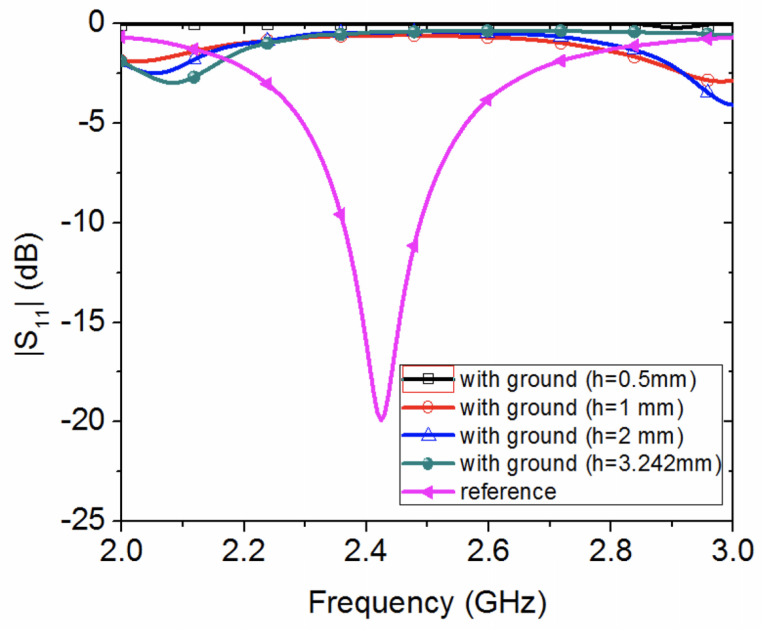
Numerical parametric study of the effect of thickness on grounded wearable loop antenna with cotton fabric.

In order to see the effect of the metallic grounded sheet on the wearable loop antenna resonance, a parametric study (see Figure 13) is numerically conducted, by varying the thickness of the textile cotton fabric between 0.5 mm to 3.242 mm. A well-matched case, labeled reference, can be seen for the ungrounded wearable loop antenna on 0.242 mm-thick cotton fabric.

## 3. Wearable Loop Antenna with HIS Structure

**Figure 14:**
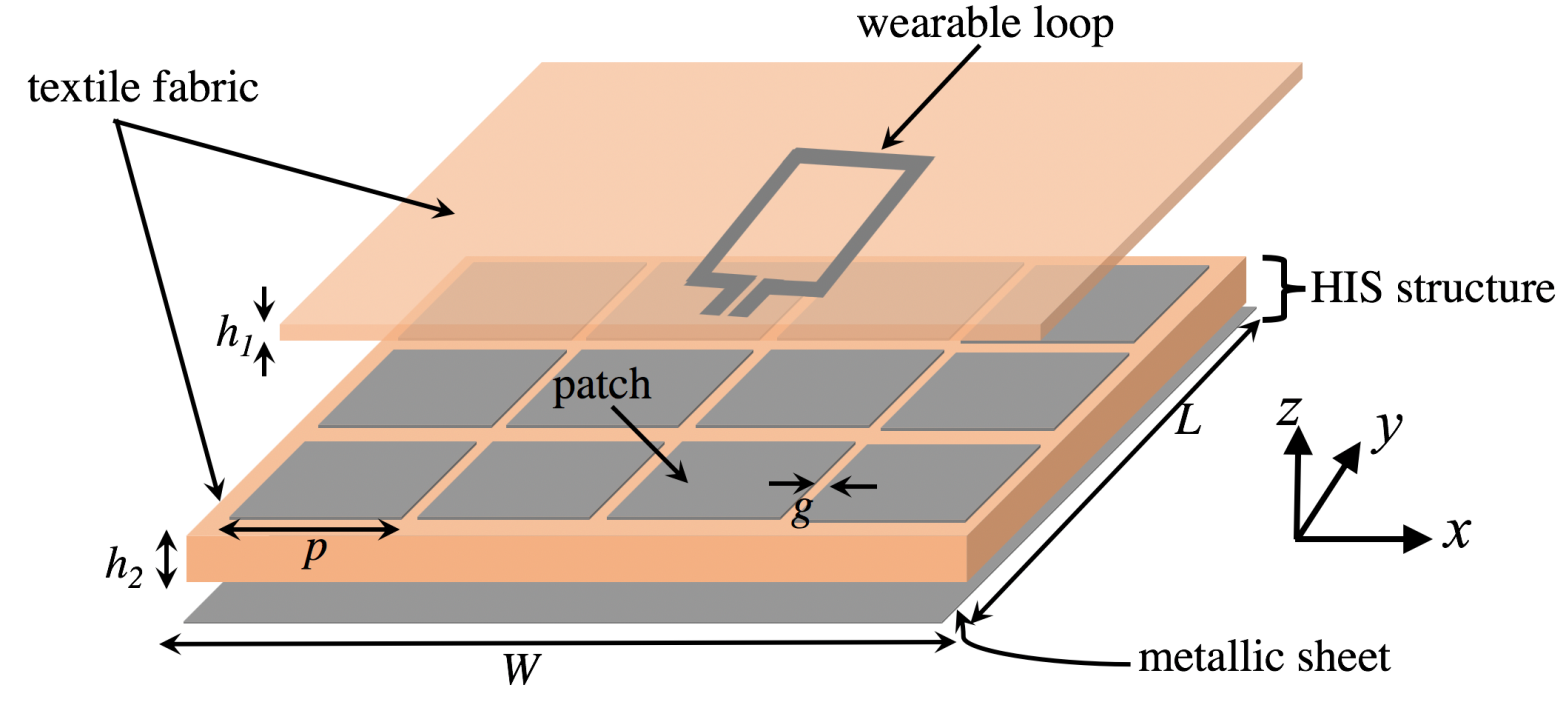
Schematic of the wearable loop antenna with high-impedance surface (HIS) structure.

In this section, the problem of wearable loop antenna performance degradation when backed with a metallic ground sheet is tackled using high-impedance structure. Figure 14 shows the proposed structure, in which the textile wearable loop antenna is placed on top of a grounded HIS structure. In order to mimick sufficient periodicity for the HIS structure, a finite-size HIS structure with 4 metallic patches along x-axis and 3 patches along y-axis is considered, resulting in 12 metallic patches. The wearable loop antenna structure is of size *L* = 145 mm, *W* = 112 mm, and an overall thickness of 3.424 mm (i.e., $h_{1}$ = 0.424 mm and $h_{2}$ = 3 mm).

The HIS structure was designed to achieve an operational in-phase reflection regime, where the 2.45 GHz-frequency band lies within the defined ±45${}^{\circ }$ reflection phase band. The HIS structure has a metallic patch of size *p* = 34 mm, gap spacing between the patches, *g* = 2 mm, with a 3-mm thick cotton fabric as its substrate material.

**Figure 15:**
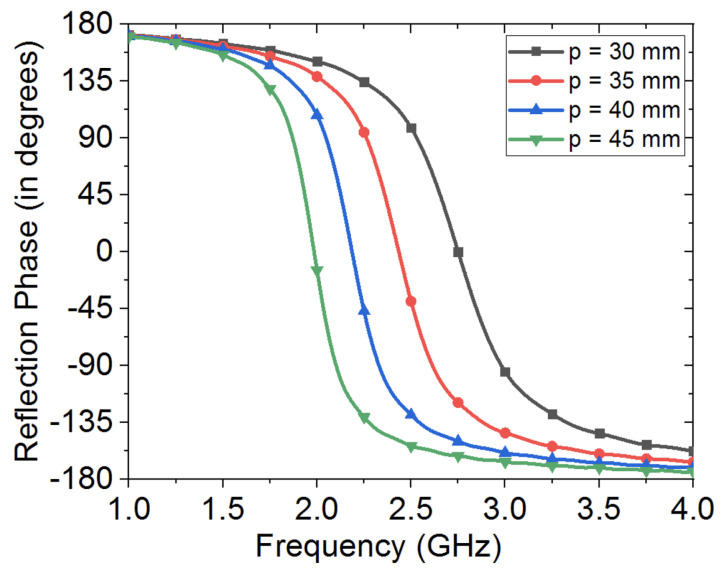
Effect of metallic patch size on operational in-phase reflection frequency band of the HIS structure. Note that periodic boundary conditions were applied in order to mimic a periodic HIS structure. Note that gap between patches, g, was kept fixed here as 2 mm.

**Figure 16:**
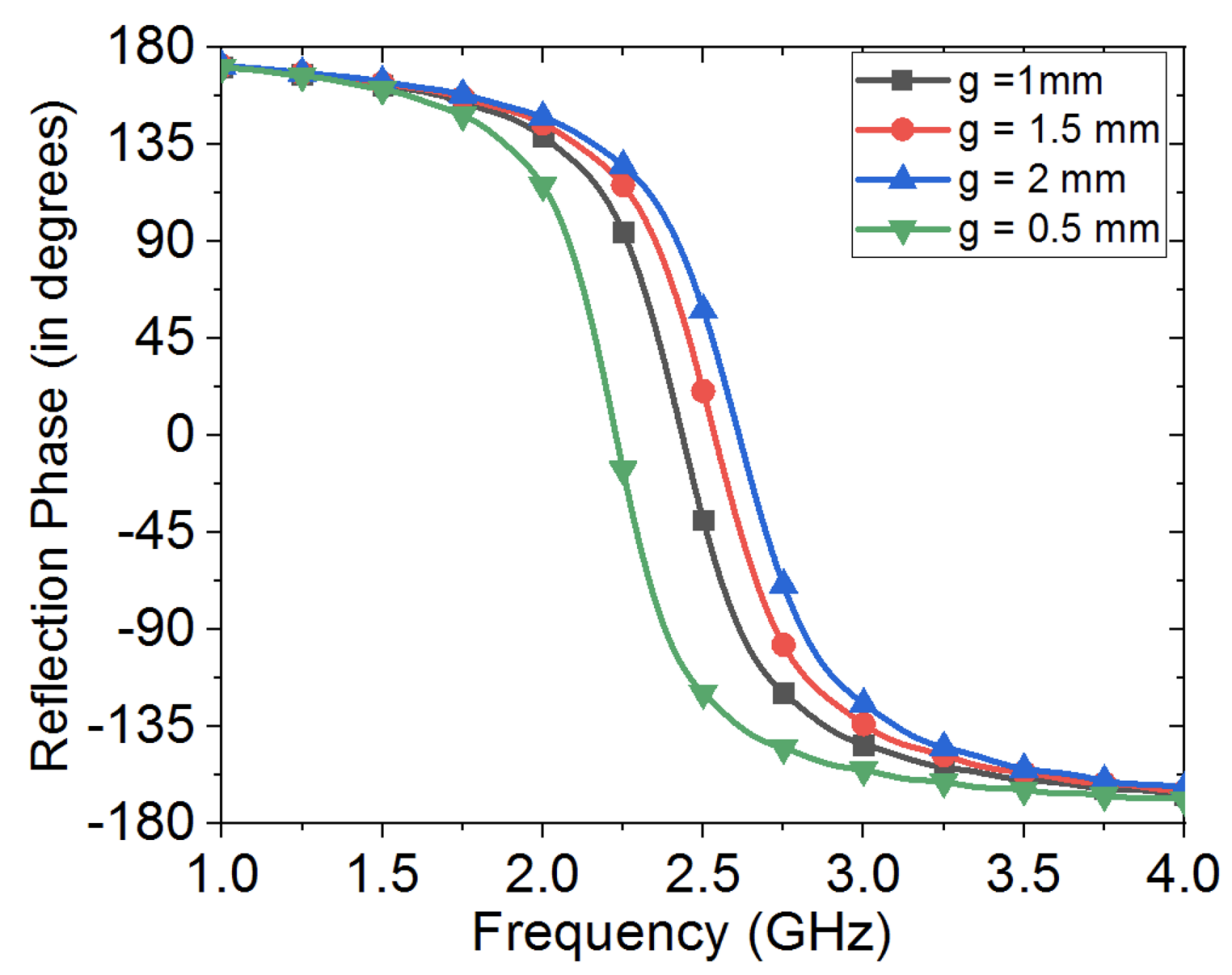
Effect of gap size on operational in-phase reflection frequency band of the HIS structure. Note that periodic boundary conditions were applied in order to mimic a periodic HIS structure. Note that metallic patch size, p, was kept fixed here as 35 mm.

Figure 15 shows the effects of varying the HIS patches size, where the 0${}^{\circ }$ in-phase can be seen to shift to higher frequencies, as metallic patches are made smaller in size, while the effect of varying the interspacing between the metallic patches of the HIS structure can be seen in Figure 16, where strong capacitive coupling results when gap between metallic patches gets smaller in size, and hence results in shifting the in-phase reflection band of the HIS towards lower frequency regime.

**Figure 17:**
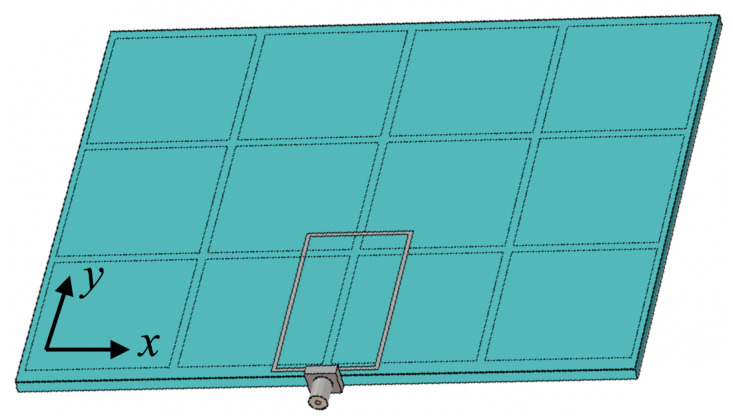
Perspective view of the modeled wearable loop antenna with HIS structure in CST MWS.

**Figure 18:**
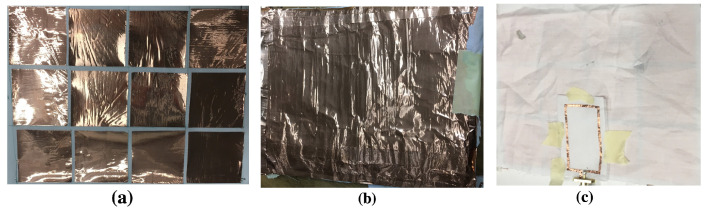
Images of the fabricated wearable loop antenna with HIS structure, (**a**) embedded HIS metallic patches, 4 × 3 elements, (**b**) HIS metallic ground plane, and (**c**) wearable loop antenna on top of HIS structure. Note that insulating tape was used to remove any air gaps between the layers.

Figure 17 shows the numerical model of wearable loop antenna with HIS structure in CST MWS, while the fabricated prototype can be seen in Figure 18. For ease of construction, adhesive copper tape was used for the construction of the HIS metallic patches and ground sheet.

**Figure 19:**
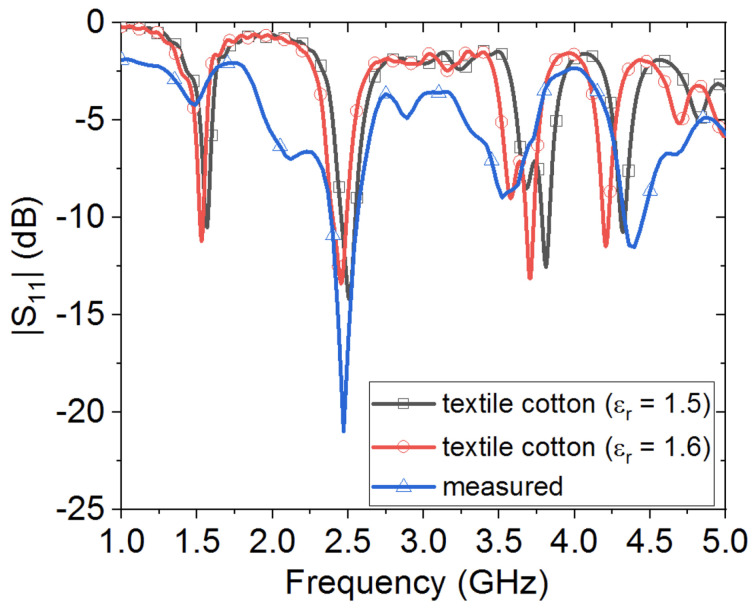
Simulated versus measured reflection coefficient results for the wearable loop antenna with the HIS structure.

Figure 19 depicts the measured reflection coefficient for the wearable loop antenna with the HIS, and compared against simulated results when using textile cotton fabric with two different electric permittivity values, 1.5 and 1.6. Good agreement between simulated and measured data can be seen, especially within the 2.45 GHz band. It was observed that the resonance dip of the reflection coefficient was at 2.47 GHz for the measured case, while it was at 2.5 GHz for the textile cotton fabric with $\epsilon_{r}$ = 1.5, and at 2.45 GHz for the case of textile cotton fabric with $\epsilon_{r}$ = 1.6.

**Figure 20:**
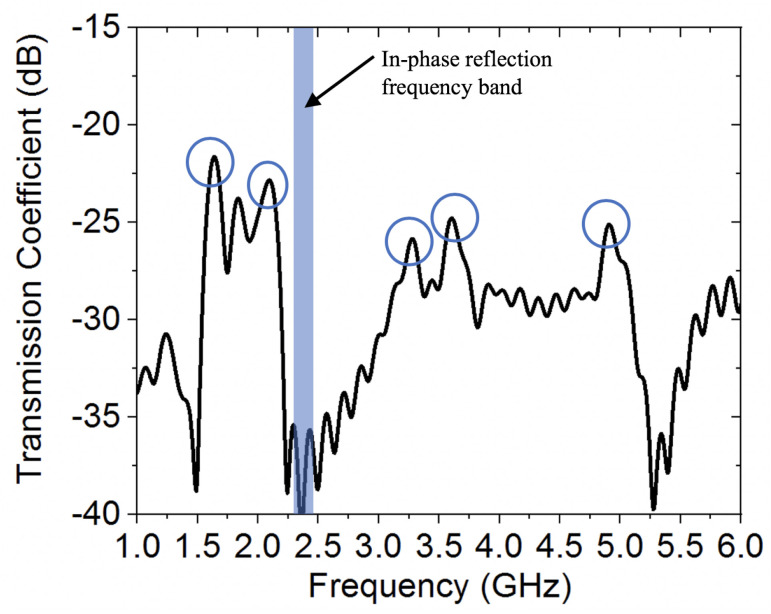
Computed transmission coefficient between the two cotton fabric layers of the HIS structure to estimate embedded parallel-plate waveguide modes.

As compared against the case of grounded wearable loop antenna, the HIS structure resulted in significant impedance improvement, due to the inherent characteristic of 0${}^{\circ }$ in-phase reflection between the low-profile wearable loop antenna and its image. Hence, maintaining good resonance behavior for the wearable loop antenna. Several additional resonances were also observed within both simulated and measured reflection coefficient curves, which are attributed to the behavior of the HIS structure as an equivalent embedded parallel-plate waveguide structure, with such resonant modes. In fact, a numerical model was also conducted in order to investigate such resonances of the HIS-like parallel-plate structure, where two ports were launched, one within the top cotton layer, with $h_{1}$ = 0.242 mm, while the second one was embedded within the second cotton fabric layer, with $h_{2}$ = 3 mm. Figure 20 shows the computed transmission coefficient, an indication of the power coupled from the top to the bottom cotton layer. As can be seen, the additional resonances in Figure 19 can be identified clearly in Figure 20.

**Figure 21:**
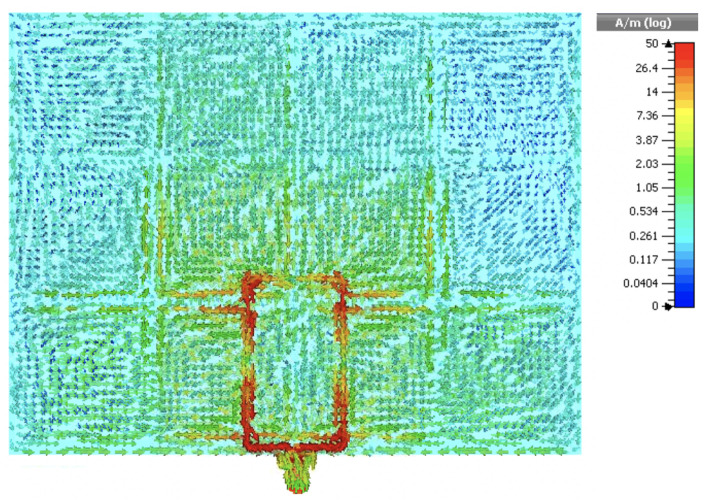
Snaphot of the electric surface current distribution at the surface of wearable loop antenna with HIS structure, computed at a frequency of 2.5 GHz.

Figure 21 depicts the surface current distribution along the wearable loop with HIS structure, plotted at a frequency of 2.5 GHz, where strong electric current density distribution is seen along the loop’s edges. Significant coupling effects are also observed between the loop antenna and the HIS metallic patches beneath the antenna, which contributed to the additional resonances in Figure 19.

**Figure 22:**
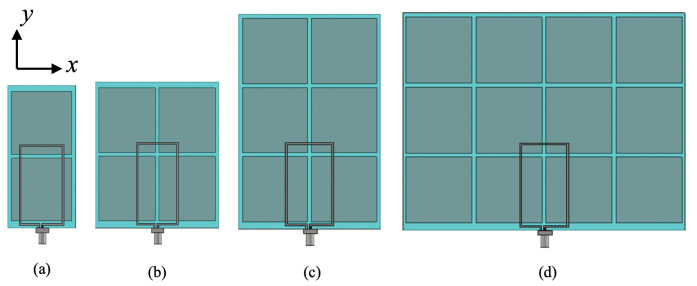
Top view of the wearable loop antenna on top of various HIS sizes: (**a**) case 1, (**b**) case 2, (**c**) case 3, and (**d**) case 4.

Another numerical study was conducted in order to investigate the performance of the wearable loop antenna when placed in a smaller HIS surface as compared against the proposed structure in Figure 17. Figure 22 depicts the numerically studied cases, labeled as case 1 (1 × 2 HIS unit cells), case 2 (2 × 2), case 3 (2 × 3), and compared against the previously studied case of 4 × 3 HIS unit cells (denoted as case 4 here). Table 1 summarizes the results. An increase in peak gain is achievable as number of HIS unit cells is increased, which is essential to have sufficient periodicity for the HIS. For instance, antenna’s simulated peak gain for case 1 was around 4.08 dBi as compared against that gain of antenna in case 4, which was 6.19 dBi. From the numerically computed radiation efficiency, the encountered losses within the textile fabric were only an estimation (tanδ = 0.02), which reflects to be higher than the adopted fabrics.

**Figure 23:**
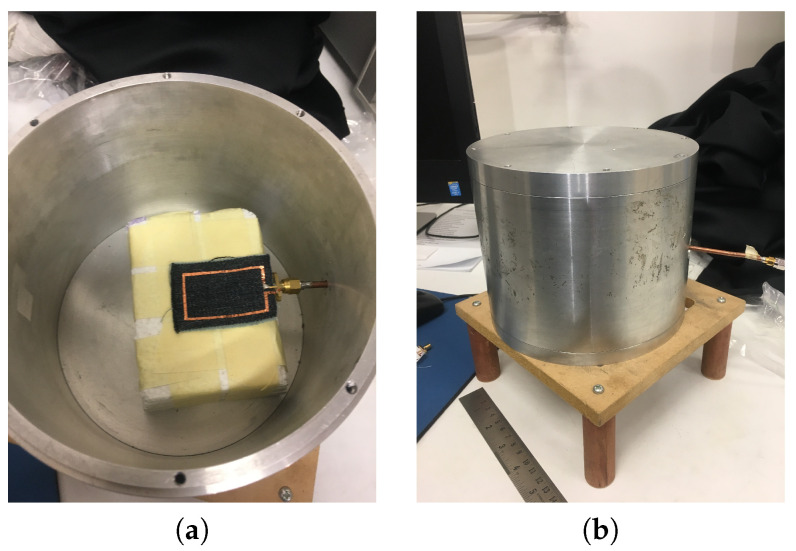
Images of the experimental setup for efficiency measurement of the textile loop sensor with and without HIS surface (case 4), (**a**) top view and (**b**) perspective view of the cylindrical-shaped wheeler cap.

Furthermore, the radiation efficiency of the wearable loop antenna with (case 4) and without HIS surface was experimentally measured using the Wheeler Cap method [29,30,31]. Figure 23 shows two images from the experimental setup for efficiency measurement. Table 2 summarizes the measured radiation efficiency of textile loop antennas with and without HIS structure. Both fabrics were considered here. As can be seen from the results, measured efficiencies of loop antenna made from wash cotton (jeans) fabric is lower than those made from cotton, which are attributed to nature of the fabric from its structure point of view. Moreover, there is a drop in efficiency of textile antennas with HIS structure (case 4) as compared against textile loop antennas alone. This is justified as follows when number of HIS unit cells are increased, this will incur additional losses within the textile fabric (substrate in case 4) in addition to the HIS metallic patches (i.e., conductors’ losses), and results in lower measured radiation efficiency as compared against cases of wearable loop antennas alone (see Table 2).

**Figure 24:**
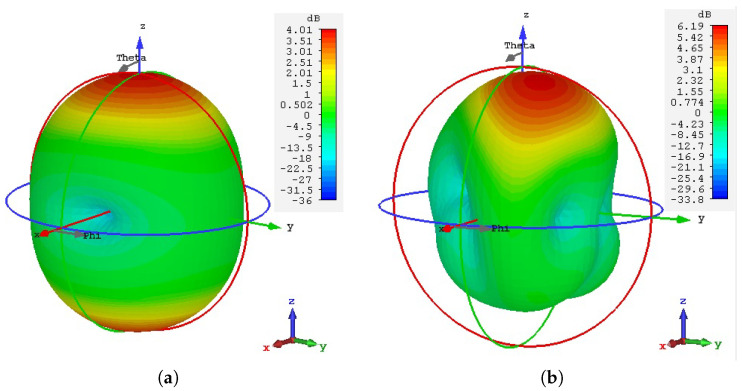
3D view of the numerically computed gain pattern for: (**a**) wearable loop antenna alone (without HIS structure); and (**b**) loop antenna with HIS structure (as shown in Figure 17).

Figure 24 depicts the 3D view of the simulated gain pattern for the wearable loop antenna with and without the HIS surface. As can be seen, most of the radiated energy is concentrated above the wearable loop antenna with HIS structure as compared against the loop antenna alone (see Figure 24a), and hence resulted in increased directivity and gain.

**Figure 25:**
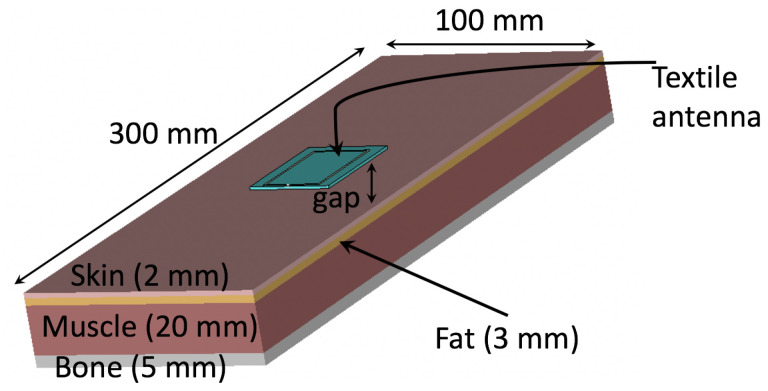
3D view of numerical model to study performance of wearable loop antenna with and without HIS in close proximity to a human arm. Note that wearable loop antenna alone (without HIS structure) is shown only.

For healthcare sensing applications, the robustnesses of the wearable loop antenna when in close proximity to human tissues need to be maintained. In this paper, the performance of the wearable loop antenna with and without the HIS structure when in close proximity to a human arm is numerically investigated next. The wearable loop antenna with the HIS structure was placed on top of a modeled phantom subject, mimicking a human arm. For comparison purposes, the performance of the wearable loop antenna without HIS structure on top of the phantom was also evaluated (see Figure 25). All electrical properties of the arm layers, comprising: skin, fat, muscle and bone have been considered from the available libraries in CST microwave studio. Figure 25 shows a 3D view of the modeled loop antenna without the HIS structure case. Parametric study was conducted to investigate the effect of varying the separation between the wearable antenna from the modeled arm, where worst case scenarios were considered, in which gaps of 1 mm and 3 mm were chosen.

**Figure 26:**
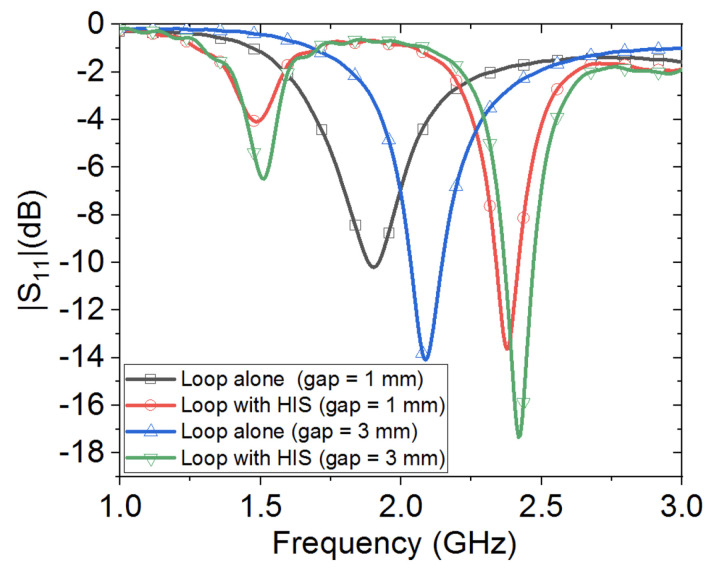
Computed reflection coefficient for the wearable loop antenna with and without HIS on top of modeled human arm with spacing of 1 mm and 3 mm.

Figure 26 shows the computed reflection coefficient for the two wearable loop antenna cases. Based on the numerical full-wave simulations, it can be observed from Figure 26 that the HIS structure had maintained good performance of the wearable loop antenna (i.e., well matched), while the wearable loop antenna without the HIS structure had resulted in major shift to its resonance frequency due to the close proximity to the highly lossy human arm tissues. For instance, the case of wearable loop antenna alone (without HIS) with spacing of only 1 mm above the modeled arm was fairly matched, within the −10 dB threshold level. Note that the small dip around the frequency of 1.5 GHz for the case of the wearable loop antenna with HIS is due to the embedded HIS structure resonances, as explained earlier. It can be concluded from this short numerical parametric study that the HIS structure is well suited for such healthcare applications, in which integration of wearable loop antenna with biological tissues is needed for continuous monitoring of certain body parts.

**Figure 27:**
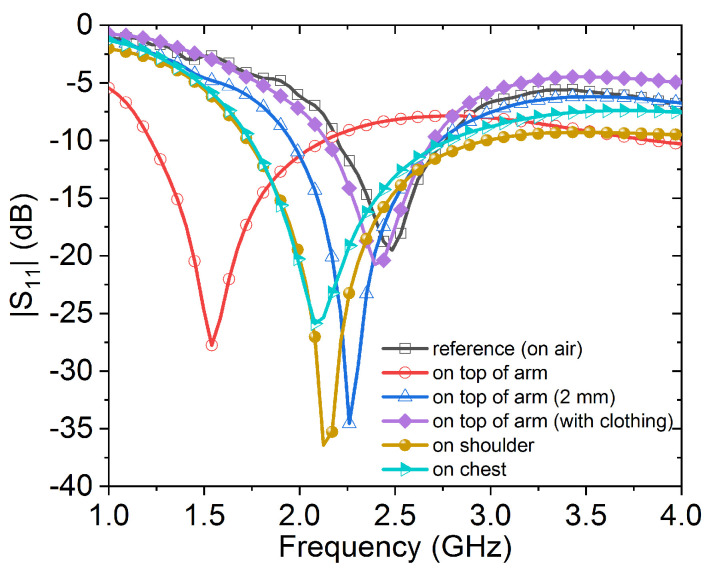
Measuredreflection coefficient for the wearable loop antenna alone when installed on various positions of a male subject.

For validation purposes, Figure 27 depicts measurements for the performance of the wearable loop sensor (cotton fabric with metallic width of 1 mm) when placed on installed on various positions of a male subject: on top of arm, shoulder and chest. Very good agreement between the measured $S_{11}$ (in Figure 27) and simulated one (in Figure 26) can be seen for the case when the wearable sensor is placed on top of arm by 2 or 3 mm.

## 4. Conclusions

In this paper, the performance of wearable loop antenna that was printed on very thin textile fabrics was assessed both numerically and experimentally. Parametric studies had been carried out to study and quantify the behavior of the antenna’s resonance as the textile fabrics’ electrical properties varied as well as their thickness. In this paper, two textile fabrics: cotton and jeans (wash cotton) were considered. For ease of construction, adhesive copper tape was used to print loop antennas on the fabrics, which is a cost-effective fabrication alternative.

Based on the numerical full-wave simulations, it was observed that as the textile properties, both textile fabrics permittivity and thickness, were increased, the wearable antenna’s resonance frequency tends to shift to lower frequencies, however, the tendency of the shift to lower frequencies is much faster for jeans fabric as compared against cotton fabric-based loop antenna. Experimental studies had also been conducted in order to investigate robustness of the wearable loop antenna when in bending conditions. Based on the results, satisfactory performance was observed, despite slight shifts on the antenna’s resonance frequency, which is attributed mainly to fabrics nature and small thickness.

For many medical applications when using wearable low-profile antennas, metallic reflectors are often employed, which then results in performance degradation of such antennas. To alleviate this problem, a low-cost flexible high-impedance surface was used as a backing structure in this work for performance enhancement of grounded wearable loop sensors. The wearable loop antenna with embedded HIS structure was designed, numerically simulated and experimentally validated. Good agreement was achieved between measured and simulated results. The effects of placing the wearable loop antenna with HIS structure in close proximity to a modeled human arm was numerically studied and compared against a wearable loop antenna without HIS structure, in which good performance was achieved for the case of the loop antenna with the HIS structure as compared against the case without HIS backing structure.

## Figures and Tables

**Table 1:** Numerical parametric study for the performance of the wearable loop antenna (cotton fabric) on top of several HIS structure sizes.

Parameter(s)	Case 1	Case 2	Case 3	Case 4
No. of HIS unit cells	1 × 2	2 × 2	2 × 3	4 × 3
Geometry dimension in mm (*x*,*y*)	(38,77)	(74,77)	(74,112)	(145,112)
Peak gain (in dBi)	4.08	4.15	4.55	6.19
Simulated Rad. efficiency (%)	31	32	58	61

**Table 2:** Comparison of measured radiation efficiency for wearable loop antennas with HIS structure as compared against ungrounded wearable loop antennas.

Parameter(s)	Loop Alone (Cotton)	Loop with HIS (Cotton)	Loop Alone (Jeans)	Loop with HIS (Jeans)
$f_{res.}$ (in GHz)	2.517	2.455	2.474	2.257
Measured Rad. efficiency (%)	89	80	77	55

## Abbreviations

The following abbreviations are used in this manuscript:

HIS	High Impedance Surface
PVC	Polyvinyl Chloride Cylinder
